# Evaluation of Serum Human Epididymis Protein 4 Levels in Women with Polycystic Ovary Syndrome

**DOI:** 10.7759/cureus.5736

**Published:** 2019-09-24

**Authors:** Necati Hancerliogullari, Aytekin Tokmak, Meryem Kuru Pekcan, Aysegul Oksuzoglu, Tuba Candar, Yaprak Ustun

**Affiliations:** 1 Obstetrics and Gynecology, Zekai Tahir Burak Women's Health Education and Research Hospital, Ankara, TUR; 2 Obsterics and Gynecology, Zekai Tahir Burak Women's Health Education and Research Hospital, Ankara, TUR; 3 Medical Biochemistry, Ufuk University, Ankara, TUR

**Keywords:** human epididymis protein 4, gynecological cancers, polycystic ovary syndrome, ovarian cancer, diagnosis

## Abstract

Aim

The main purpose of this study is the determination of serum epididymis protein 4 (HE4) levels in women diagnosed with polycystic ovary syndrome (PCOS) and comparison with non-PCOS healthy controls.

Methods

All consecutive women, who applied between January 2017 and June 2017 to the gynecology outpatient clinics at the Zekai Tahir Burak Women's Health Training and Research Hospital and met the study criteria, were included in this cross-sectional study. Serum human epididymis protein 4 (HE4) concentrations were measured in each woman and the mean values were compared between the PCOS and non-PCOS groups.

Results

A total of 90 women (45 with PCOS and 45 without PCOS) were included in the final analysis. There were no statistically significant differences between the groups in terms of age and body mass index (*p* >0.05). Basal serum HE4 levels were 172.8 ± 139.8 and 131.8 ± 123.1 pmol/L in the PCOS and non-PCOS groups, respectively (*p* = 0.415).

Conclusion

The serum HE4 levels were found to be similar in women with and without PCOS. No significant correlation was observed between PCOS parameters and serum HE4 levels.

## Introduction

Polycystic ovary syndrome (PCOS) is a clinical condition primarily characterized by ovulatory dysfunction and hyperandrogenism. Although the diagnostic criteria are constantly reviewed and updated, it is reported to be the most common and most important endocrine disorder affecting women of reproductive age with a prevalence ranging from 5% to 18% [1]. In the long term, these patients are at risk for infertility, metabolic syndrome and type 2 diabetes, and other adverse health conditions such as cardiovascular diseases and endometrial cancer. In addition, epidemiologic studies have shown that women with PCOS have a higher risk of developing ovarian cancer (odds ratio: 2.5, 95% confidence interval 1.1-5.9) [2]. Some biomarkers such as calreticulin, malate dehydrogenase, superoxide dismutase, fibrinogen-g, vimentin, and lamin B2 have been shown to increase in both PCOS and ovarian cancer women, and these markers have been reported to help improve our understanding of the linkages between PCOS and ovarian cancer [3]. Furthermore, the researchers conducting these studies claimed that such biomarkers could be used to identify subgroups of women with PCOS who are at potentially increased ovarian cancer risk.

Human epididymal protein 4 (HE4) is a secretory protein first identified in the distal epithelial cells of human epididymal channels by Kirchhoff [4]. The molecular structure of HE4 is similar to proteinase inhibitors and has a function in sperm maturation [4-5]. In the female genital system, it is expressed in the endocervical and endometrial glands, fallopian tubes, and Bartholin glands. It was also detected in the epithelium of the ovarian cortical inclusion cysts. HE4 has primarily a high diagnostic value in tumors of Mullerian origin. High serum HE4 levels have been investigated extensively and have been identified as an essential biomarker for ovarian cancer, particularly in the differentiation of benign and malignant ovarian tumors [6-7].

The serum HE4 level has been recognized by the US Food and Drug Administration as an important marker in the diagnosis of ovarian cancer since 2008. It is now used as a potential tumor marker particularly for the epithelial ovarian cancers [8-9]. HE4 has a higher diagnostic value in serous and endometrioid subtypes, which also constitute a large proportion of epithelial ovarian cancers, but other malignancies may also lead to increased HE4 levels [10]. It has a positive clinical value in three-quarters of endometrial cancer patients, but it is not surprising because it is normally present in the endometrium. Bignotti et al. found higher HE4 levels in endometrial cancer patients compared to women with normal endometrium [11]. Moreover, higher HE4 levels were observed in the aggressive endometrial carcinoma phenotype. Therefore, it may be beneficial in the follow-up of endometrial cancer which is of clinically high risk and will receive aggressive adjuvant therapy. High HE4 levels can also be seen in primary tubal cancers [12]. In addition, patients with lung adenocarcinomas and transitional cell carcinomas were found to have higher serum HE4 levels than patients with benign lesions and/or healthy individuals [6,12-13].

In this study, we measured serum HE4 levels in women diagnosed with PCOS and compared them with non-PCOS healthy controls. Thus, we aimed to test whether serum levels of HE4, an important biomarker for ovarian cancer and have never been studied in women with PCOS, are increased or not in those patient group.

## Materials and methods

PCOS patients who attended to the gynecology outpatient clinics of the Zekai Tahir Burak Women's Health Research and Education Hospital, Ankara, Turkey were registered to the present study prospectively between January 2017 and June 2017. The local ethics committee of the current hospital approved the study, and each woman gave written and verbal informed consent before they enrolled in the study. A total of 45 patients, who were diagnosed with PCOS according to the ESHRE/ASRM proposal introduced in 2004 and met at least any two of three criteria declared in the consensus statement, formed the study group. Age and body mass index (BMI) matched 45 patients free from PCOS were selected as the controls [14]. Skin (BMI <18) and obese (BMI >30) patients as well as adolescents (age <19) and patients over 40 years of age, women with autoimmune disease, those with acute and/or chronic inflammatory disease, hepatic and renal failure, and any hematological disorders were excluded from this study. Patients having other endocrinological diseases that conflict with PCOS were also excluded from the study. All control patients did not meet any diagnostic criteria used for PCOS.

Oligomenorrhoea was defined as the presence of excessive cycle length (>45 days) or less than eight spontaneous menstrual cycles per year, and amenorrhea was the absence of menstruation for more than three consecutive months. The ovaries were considered polycystic by transvaginal ultrasonography when increased stromal echogenicity was peripherally surrounded by more than 12 follicles with a diameter of 2-8 mm or an ovarian volume of >10 cm^3^ detected on at least one of the ovaries [15]. The ultrasonographic evaluations were performed by the same investigator with the same ultrasound machine for all subjects. Clinical hyperandrogenism was considered as Ferriman Gallwey score >8, whereas biochemical hyperandrogenism was accepted when total testosterone (TT) and/or dehydroepiandrostenedione sulfate (DHEA-S) level was >95th percentile of controls [16].

Laboratory studies**

Laboratory studies were performed at the 2nd or 3rd day of a spontaneous or progesterone-induced menstrual cycle for each subject on an empty stomach in the early morning. Biochemical, hormonal, and hematologic evaluations consisted of fasting glucose and insulin, total cholesterol (T-C), low-density lipoprotein cholesterol (LDL-C), high-density lipoprotein cholesterol (HDL-C), triglycerides (TG), C-reactive protein (CRP), follicle-stimulating hormone (FSH), luteinizing hormone (LH), estradiol (E2), free-T, 17-hydroxyprogesterone (17OH-P), dehydroepiandrostenedione sulfate (DHEA-S), prolactin (PRL), thyroid-stimulating hormone (TSH), and complete blood count parameters (Table 2).

After overnight fasting, blood samples were taken and instantly centrifuged at 4000 rpm for 10 min. Serum biochemistry including serum glucose was determined using the glucose hexokinase method (Roche Hitachi Cobas 6000, Roche Diagnostics GmbH, Germany). Baseline hormone levels including FSH, LH, E2, TSH, PRL, total testosterone, DHEA-S, and insulin were measured using an electrochemiluminescence immunoassay (ECLIA; Roche Cobas e 601, Roche Diagnostics GmbH, Germany).

Levels of T-C and TG were determined with enzymatic colorimetric assays (Beckman Coulter Fullerton, CA, USA). HDL-C and LDL-C concentrations were determined by a colorimetric method (Beckman Coulter Fullerton, CA, USA). CRP was measured by a nephelometric method using the BN II System (Siemens, Erlangen, Germany). The presence of insulin resistance was investigated using basal fasting insulin concentrations, fasting glucose concentrations and homeostasis model assessment-insulin resistance (HOMA-IR). HOMA-IR was calculated using the formula fasting glucose (mmol/l) x fasting insulin (mIU) x 0.055/22.5. Insulin resistance was defined as an abnormal result in HOMA index >2.5 (16).

Blood samples were taken to plain red biochemistry tube for HE4 analysis and centrifuged at 4000 rpm for 12 minutes in a refrigerated centrifuge. Serum was separated and stored at -80 °C until the assay day. On the working day, all sera were gradually thawed in the refrigerator. Measurement of serum HE4 levels was performed using a UT6100 auto microplate reader (MRC Lab., Holon, Israel) using the HE4 ELISA Kit (Sunred Biological Technology Co., Ltd., Shanghai, China). Results were reported as pmol / L. Both inter- and intra-assay coefficients of variations were less than 10% for this measurement.

Statistical analysis**

The data were analyzed with SPSS version 22.0 for Windows. The normal distribution suitability of the data was examined by the single sample Sapiro Wilk test and homogeneity of variance was evaluated by using Levene’s test. Statistical studies were expressed as mean ± standard deviation or number (percent). In the intergroup comparisons, independent *t*-test was used for normally distributed variables and Mann-Whitney U test for non-normal distribution. Spearman correlation analysis was used to examine inter-variable correlations. Statistical hypotheses were evaluated according to the two-tailed perception rate. A *p*-value <0.05 is considered statistically significant.

## Results

The present study was conducted on a total of 90 patients with and without PCOS. There were 45 patients in the PCOS group and 45 age and BMI-matched healthy women in the control group. The mean age of patients with PCOS was 28.7 ± 5.3 years, and the mean age of control patients was 29.3 ± 5.5 years (*p* = 0.145). The mean values of BMI, WC, HC, and WHR of the groups were similar (all *p* > 0.05). The demographic and anthropometric features of the cases are shown in Table 1.

**Table 1:** Clinical parameters in PCOS in patients and controls PCOS, polycystic ovary syndrome; BMI, body mass index; WC, waist circumference; HC, hip circumference; WHR, waist to hip ratio. FGS, Ferriman Gallwey Score; data are presented as mean ± standard deviation. P <0.05 is considered statistically significant.

Variables	PCOS (n: 45)	Control (n: 45)	P
Age (years)	28.7 ± 5.3	29.3 ± 5.5	0.145
BMI (kg/m^2^)	25.2 ± 3.7	24.7 ± 3.2	0.194
WC (cm)	84.6 ± 7.6	80.2 ± 8.4	0.176
HC (cm)	100.3 ± 6.1	100.2 ± 5.7	0.455
WHR	0.83 ± 0.07	0.82 ± 0.07	0.348
FGS	12.2 ± 6.1	5.3 ± 3.8	<0.001

Baseline hormone levels, biochemical markers of glucose and lipid metabolism, and some inflammatory markers are depicted in Table 2. 

**Table 2:** Comparison of the biochemical and endocrinologic parameters in the groups PCOS, polycystic ovary syndrome; FBG, fasting blood glucose; FBI, fasting blood insulin HOMA-IR, homeostasis model assessment-insulin resistance; HDL-C, high-density lipoprotein- cholesterol; TG, triglyceride; LDL-C, low-density lipoprotein-cholesterol; Total-C, total cholesterol; LH, luteinizing hormone; FSH, follicle-stimulating hormone; E2, estradiol; TSH, thyroid-stimulating hormone; PRL, prolactin; DHEAS, dehydroepiandrostenedione sulfate; Total-T, total testosterone; 17OH-P, 17 hydroxyprogesterone; HE4, human epididymis protein 4; data are presented as mean ± standard deviation; *P *< 0.05 is considered statistically significant.

Variables	PCOS (n: 45)	Control (n: 45)	P
FBG (mg/dL)	89.0 ± 5.6	86.1 ± 6.6	0.025
FBI (mU/L)	13.8 ± 6.7	5.1 ± 3.2	<0.001
HOMA-IR	3.1 ± 2.3	1.7 ± 0.5	<0.001
HDL-C (mg/dL)	61.6 ± 7.9	64.5 ± 8.0	0.248
TG (mg/dL)	104.2 ± 54.6	102.2 ± 53.1	0.762
LDL-C (mg/dL)	74.2 ± 32.1	63.7 ± 23.8	0.112
Total-C (mg/dL)	158.1 ± 36.1	145.1 ± 29.6	0.048
LH (U/L)	9.7 ± 6.9	7.3 ± 6.1	0.036
FSH (U/L)	6.5 ± 1.8	7.1 ± 1.9	0.101
E_2 _(pg/mL)	39.3 ± 11.1	38.7 ± 15.3	0.812
TSH (U/L)	2.3 ± 1.0	2.2 ± 1.0	0.865
PRL (ng/mL)	16.7 ± 9.1	15.3 ± 7.3	0.096
DHEAS (mg/dL)	367.9 ± 176.1	329.1 ± 150.1	0.246
Total-T	0.6 ± 0.2	0.4 ± 0.2	<0.001
17OH-P(ng/ml)	1.4 ± 1.2	1.3 ± 0.8	0.332
HE4 (pmol/L)	172.8 ± 139.8	131.8 ± 123.1	0.415

Baseline serum LH levels were statistically significantly higher in the PCOS group when compared to control group (9.7±6.9 vs. 7.3 ± 6.1 U/L, p = 0.036). The mean HOMA-IR and total testosterone levels were statistically significantly higher in the PCOS group than in the control group (both *p* <0.001). The median levels of basal serum HE4 levels were 92.8 (3.6-466.7) and 71.3 (22.9-446.5) pmol/L (*p* = 0.415) in PCOS and non-PCOS groups, respectively. Spearman’s correlation analysis showed that no significant correlation was present between serum HE4 concentrations and other continuous variables in both groups (Table 3).

**Table 3:** Spearman’s rank correlation of serum HE4 levels with other variables HE4, human epididymis protein 4; PCOS, polycystic ovary syndrome; BMI, body mass index; FGS, Ferriman Gallway Score; LH, luteinizing hormone; HOMA-IR, homeostasis model assessment-insulin resistance; Total-C, total cholesterol; DHEAS, dehydroepiandrostenedion sulfate; Total-T, total testosterone; r, correlation coefficient.

Group	PCOS		Control		Total	
Variables	r	p	r	p	r	p
Age	-0.171	0.132	-0.087	0.593	-0.153	0.354
BMI	0.224	0.052	0.233	0.171	0.321	0.057
FGS	0.101	0.341	-0.057	0.704	0.112	0.476
LH	0.136	0.227	-0.027	0.860	0.289	0.087
HOMA-IR	0.204	0.062	0.148	0.327	0.280	0.084
Total-C	-0.140	0.222	-0.203	0.214	-0.087	0.599
DHEA-S	0.096	0.577	-0.001	0.994	-0.103	0.581
Total-T	0.074	0.493	-0.157	0.297	0.189	0.224

## Discussion

PCOS is a common endocrinopathic condition that occurs in the reproductive period and causes some long-term health problems [17-18]. It is characterized by signs of hyperandrogenism such as hirsutism and acne with oligo-/amenorrhea. Insulin resistance, obesity, elevated androgen concentrations, and unopposed estrogen levels are pathophysiological features. There is a high prevalence of obesity in PCOS as well as the risk of metabolic syndrome and cardiovascular diseases [14]. The link between unopposed estrogen commonly seen in PCOS patients and endometrial cancer is well established [19]. However, its relationship with other cancers, especially ovarian cancer, has not yet been clearly established. In this study, we aimed to evaluate serum HE4 concentration which is a well-known tumor marker for ovarian cancer in women with PCOS to test whether there is an association between them. There are no well-defined reference values of HE4 for women as well as for men. There is scarce clinical data regarding HE4 levels and its association with serum androgens in men. Herein, we also hypothesized that serum HE4 levels might be regulated by androgens, thus we also evaluated the possible correlation of HE4 with total testosterone. According to our study, serum HE4 levels have no diagnostic value for PCOS and have no correlation with PCOS parameters.

Chronic anovulation, prolonged exposure to unopposed estrogen leads to endometrial hyperplasia that was followed by endometrial cancer. Risk factors for endometrial cancer are obesity, hypertension, type 2 diabetes, unopposed estrogen, and nulliparity. All of these conditions are also associated with PCOS. Again, elevated levels of estrogen can cause other hormone-sensitive tumors to grow and can trigger some cancers such as breast and ovarian cancer. Hormones and growth factors cause malignant changes in ovarian epithelium. Berchuck et al. claimed that those changes increase the risk of ovarian cancer in PCOS [20]. This was supported by another study conducted by Rao and Slotman showing the relationship between PCOS and gynecological cancers [21]. The relationship between endometrial cancer and PCOS is already well known [22]. In the meta-analysis performed by Barry JA et al. about the incidence of gynecologic malignancies in PCOS patients, the risk of endometrial cancer was found to be higher in women of all ages, but this increase was not observed in ovarian and breast cancer [23]. Although some previous studies have suggested an increased risk of ovarian cancer in PCOS, in a recently published large epidemiological study, some genes associated with PCOS were examined in patients with invasive ovarian cancer and found a lower risk of invasive ovarian cancer in patients with genetically predisposed to PCOS [24]. The authors assessed that obesity, which is a common risk factor for ovarian cancer and a confounding factor in PCOS, is ignored or not adjusted in the previous studies. They explained the opposed results between the studies in this way.

Some tumor markers may elevate in numerous benign and malignant conditions. The serum CA125 level is one of the most useful biomarkers in the follow up after treatment of ovarian cancer, but CA125 level alone itself is not a useful screening marker for ovarian malignancies. In a large study, serum HE4 level was found to be an important tumor marker in differentiating between ovarian cancer and benign gynecologic conditions and serum HE4 level was suggested to be as important as CA125 in the diagnosis of ovarian cancer [9]. There is only one study investigating CA125 levels in patients with PCOS, and in that study, it was found that Ca125 increased significantly in PCOS [25]. In addition, LH and prolactin levels were found to be higher in women with PCOS than non-POCS women. It has been claimed that the combination of CA125 with this hormonal condition may be a biochemical screening method for ovarian cancer. Of course, this claim is quite ambitious with this sample size and study design.

As far as we know, our study is the first to evaluate HE4 levels in patients with PCOS. However, the limited number of patients and the absence of a PCOS group with ovarian cancer are important limitations. In order to claim that HE4 is a potential marker of early ovarian cancer in patients with PCOS, this very young group of patients should be followed up on a long-term basis. Further well-designed large-scale prospective studies are needed to determine whether HE4 is a risk factor for both PCOS and ovarian cancer. However, at this point, another difficulty in evaluating the association of HE4 with PCOS and ovarian cancer is that these heterogeneous diseases have different subgroups.

## Conclusions

Given the possible risk of developing gynecologic cancers, particularly endometrium and ovarian cancer, in patients with PCOS, in this study, we examined whether HE4, which has a diagnostic value for ovarian cancer, can be used as a potential diagnostic biomarker for PCOS and is an alarming indicator of ovarian cancer risk in those patients. According to the results, there was no significant difference between the PCOS and non-PCOS groups in terms of serum HE4 levels. In summary, PCOS was not associated with serum HE4 levels. Further studies are needed to evaluate the relationship between PCOS and HE4.
